# In Vitro Fecal Fermentation Patterns of Arabinoxylan from Rice Bran on Fecal Microbiota from Normal-Weight and Overweight/Obese Subjects

**DOI:** 10.3390/nu13062052

**Published:** 2021-06-15

**Authors:** Inah Gu, Wing Shun Lam, Daya Marasini, Cindi Brownmiller, Brett J. Savary, Jung Ae Lee, Franck Carbonero, Sun-Ok Lee

**Affiliations:** 1 Department of Food Science, Division of Agriculture, University of Arkansas, Fayetteville, AR 72704, USA; inahgu@uark.edu (I.G.); wshunlam@gmail.com (W.S.L.); dnm385@gmail.com (D.M.); cbrownm@uark.edu (C.B.); 2 Arkansas Biosciences Institute, Arkansas State University, Jonesboro, AR 72467, USA; bsavary@astate.edu; 3 College of Agriculture, Arkansas State University, Jonesboro, AR 72467, USA; 4 Agricultural Statistics Laboratory, Division of Agriculture, University of Arkansas, Fayetteville, AR 72704, USA; julee@uark.edu; 5 Department of Nutrition and Exercise Physiology, Washington State University, Spokane, WA 99202, USA; franck.carbonero@wsu.edu

**Keywords:** arabinoxylan, gut microbiome, rice bran fiber, short-chain fatty acids

## Abstract

Arabinoxylan (AX) is a structural polysaccharide found in wheat, rice and other cereal grains. Diets high in AX-containing fiber may promote gut health in obesity through prebiotic function. Thus, the impact of soluble AX isolated from rice bran fiber on human gut microbiota phylogenetic composition and short-chain fatty acid (SCFA) production patterns from normal-weight and overweight/obese subjects was investigated through in vitro fecal fermentation. Results showed that rice bran arabinoxylan modified the microbiota in fecal samples from both weight classes compared to control, significantly increasing *Collinsella*, *Blautia* and *Bifidobacterium*, and decreasing *Sutterella*, *Bilophila* and *Parabacteroides*. Rice bran AX also significantly increased total and individual SCFA contents (*p* < 0.05). This study suggests that rice bran AX may beneficially impact gut health in obesity through prebiotic activities.

## 1. Introduction

The gut microbiota have received extensive attention in the last two decades because of their important role in host health and gut homeostasis [1]. Gut microbiota is the collective term of microorganisms living in the host gut, providing many health benefits to the host, including gut integrity and immune system improvement, energy harvest and protection from pathogens [2,3,4,5]. However, the gut microbiota are also related to many diseases, and the composition of the gut microbiota is known to be affected by diseases such as obesity [6,7], type 2 diabetes mellitus [8,9], cardiovascular diseases [10,11] and colon cancer [12,13].

Obesity is caused by excessively accumulated body weight from an imbalance between energy intake and expenditure, mostly due to consumption of highly processed foods high in fat and sugar and a sedentary lifestyle [14]. Obesity is a leading public health issue in the US, affecting 42.4% of US adults [15]. It is a proven risk factor for many other diseases, such as type 2 diabetes [16,17], hypertension [18], cardiovascular diseases [19] and certain cancers [20,21]. Many studies have associated gut microbiota and diet with obesity, identifying changes in the composition of gut microbiota through different diets leading to weight loss or obesity; thus, the gut microbiota and diet might be a good target for obesity studies [22,23,24,25,26,27].

The consumption of dietary fibers has garnered interest as prebiotics for gut microbiota [28,29,30]. Dietary fiber consists of polysaccharides that cannot be hydrolyzed by human digestive enzymes. Dietary fiber can be fermented by gut microbiota to short-chain fatty acids (SCFAs), which improve the intestinal environment directly or indirectly by lowering the pH, crossing the epithelial barrier and metabolizing butyrate primarily by epithelial cells, propionate by liver cells and acetate by muscles after uptake and use by host cells [31,32]. SCFAs have an important role in gut homeostasis with further impact on host health and metabolism [33]. SCFAs include acetic acid, propionic acid and butyric acid at a 60:20:20 ratio, with a total concentration of 50–200 mM [34]. SCFAs have distinguishable impacts on the host metabolism [35].

Arabinoxylans (AXs) are polysaccharides composed of a β-1,4-xylan polymer substituted predominantly with arabinose side chains [36]. AXs are major structural cell wall components in cereals (maize, rye, barley, oats, rice, sorghum and wheat) and other plants, such as bananas [37]. They are extractable with alkali from cereal brans, which are byproducts of grain processing [38]. Numerous studies have examined the impact of maize, wheat and rye arabinoxylans on gut microbiota, but the impact of arabinoxylans from rice bran on human gut microbiota with regard to obesity has not been well described [39,40,41]. The prebiotic function of cereal AXs has not been investigated as extensively compared to other well-established plant dietary fibers, such as fructans (including fructooligosaccharides, FOS), galactans and β-glucans [39,42,43,44].

In vitro fecal fermentation models have been widely used to evaluate the effect of substrate on the composition and diversity of gut microbiota without ethical constraints [45]. Since human and animal studies are more laborious and expensive, and there is still a limitation regarding animal models that cannot reflect the complex human digestive system; in vitro models have been useful tools, with fecal samples representing the distal large intestine [46], but the limitation of in vitro models is that the experiments are short-term because they cannot mimic the colorectal environment and might suffer from a lack of substrates. In addition, there is a possibility to change the batch conditions by accumulated end products, causing a negative impact on their relevance with in vivo long-term studies [45]. Nevertheless, in vitro models are good for the initial screening of different substrates [46].

Thus, the objective of this study was to investigate the impact of arabinoxylan isolated from rice bran (compared with FOS as prebiotic control) on modulating the gut microbiota populations and short-chain fatty acid (SCFA) production levels through in vitro fecal fermentation from normal-weight (NW) and overweight/obese (OO) subjects.

## 2. Materials and Methods

### 2.1. Materials

Rice bran was a gift from Riceland Foods (Stuttgart, AR, USA), and FOS was obtained from Megazyme International Ireland Ltd. (Wicklow, Ireland). For fermentation medium, yeast extract and resazurin from Alfa Aesar (Ward Hill, MA, USA), peptone from Fisher Scientific (Waltham, WA, USA), bile salts from Oxoid (Hampshire, UK), sodium bicarbonate, sodium chloride, potassium hydrogen phosphate, magnesium sulfate heptahydrate, calcium chloride hexahydrate, L-cysteine hydrochloride, vitamin K, bovine hemin and Tween 80 from Sigma (St. Louis, MO, USA) were purchased. SCFA standards (acetic acid, propionic acid and butyric acid) were purchased from MilliporeSigma Corporate (St. Louis, MO, USA).

### 2.2. Isolation of Arabinoxylan

Rice bran fiber was prepared by de-starching defatted commercial bran based on a modified protocol from Bunzel et al. [47]. A total of 50 g of bran suspended in 500 mL water was heated at 90 °C for 15 min, cooled to 55 °C and digested overnight at pH 5.0 with combined 2.5 mL alpha-amylase (Fungamyl 800 L, Sigma-Aldrich, St. Louis, MO, USA) and 2.5 mL amyloglucosidase (AMG 300 L, Sigma-Aldrich) under constant mixing in a water bath shaker at 55 °C. The suspension was adjusted to pH 7.5 and further digested with 2.5 mL subtilisin A (Alcalase 2.4 L, Sigma-Aldrich) for 2 h at 55 °C. The fiber was separated by centrifugation, washed extensively with water (centrifuging and decanting until supernatant clear) and then treated overnight with 250 mL 0.25% EDTA (*w*/*v*) at 75 °C. The suspension was cooled and water washing repeated. Finally, the fiber was sequentially extracted with 100 mL of ethanol (twice) then acetone before drying by evaporation in a fume hood and vacuum desiccator.

Arabinoxylan as hemicellulose B fraction was isolated from rice bran fiber after modification from Saulnier et al. [48]. A total of 20 g fiber was extracted for 2 h at 30 °C in 200 mL of 0.5 M sodium hydroxide with 0.2% sodium borohydride, and the supernatant was separated by centrifugation. The pellet was washed with 200 mL water, and the supernatant was combined with the NaOH extract. The pellet was further extracted by 200 mL of 1.2 M potassium hydroxide with 0.5% sodium borohydride with mixing at 90 °C for 2 h and then washed with 200 mL water. The combined supernatants from treatments were combined and neutralized with glacial acetic acid, treated with 10% loading of the starch-hydrolyzing enzymes for 2 h at room temperature, then reduced to about 100 mL by rotary evaporation and finally acidified to pH 4.5. After overnight incubation at room temperature, the solution was clarified by centrifugation at 15,000× *g* for 30 min and treated with 400 mL of ethanol to recover the arabinoxylan. The composition as arabinoxylan was confirmed by the method of Blakeney et al. [49], with nominal (<3%) residual glucose.

### 2.3. Subjects and Fecal Sample Collection

This study was approved by the University of Arkansas Institutional Review Board (IRB #13-09-080). Subjects aged 21–50 from the University of Arkansas and North West Arkansas area were recruited. During the screening process, subjects filled out consent and screening forms and food frequency questionnaires (Genesis R&D analysis software, Salem, OR). Their height, weight and fasting blood glucose level (FBG) were measured. From the screening, 13 subjects with no digestive diseases, fasting blood glucose level (FBG) lower than 100 mg/dL to exclude those with prediabetes or diabetes, no tobacco use, no current medication intake and no antibiotics taken in the past 6 months were selected. They were grouped as 6 normal weight (NW) with body mass index (BMI) < 25 and 7 overweight/obese (OO) with BMI ≥ 25. A fecal sample was collected from each subject in a tightly closed bag using stool collection kits (Commode Specimen Collection System, Fisher Scientific, Waltham, WA, USA). The fecal sample was delivered within 1 h of defecation and immediately transferred and processed in an anaerobic chamber (Coy Laboratory Products Inc., Grass Lake, MI, USA) for the in vitro experiment.

### 2.4. In Vitro Human Fecal Fermentation

In vitro fecal fermentation was carried out as previously described [50]. Fecal fermentation medium (1 L) consisted of yeast extract (2 g), peptone (2 g), bile salts (0.5 g), sodium bicarbonate (2 g), sodium chloride (0.1 g), potassium hydrogen phosphate (0.08 g), magnesium sulfate heptahydrate (0.01 g), calcium chloride hexahydrate (0.01 g), L-cysteine hydrochloride (0.5 g), vitamin K (10 μL), bovine hemin (50 mg), Tween 80 (2 mL) and 0.025% (*w*/*v*) resazurin solution [51]. After bubbling the medium in test tubes with nitrogen gas (N_2_) for 3 h, the tubes were sealed with a rubber stopper and autoclave tape and autoclaved at 121 °C for 15 min. All following procedures were carried out in an anaerobic chamber. In an anaerobic chamber, the fecal sample (2 g) was mixed with 20 mL of phosphate-buffered saline until homogeneous and filtrated through 4 layers of cotton gauze to prepare the fecal slurry. In test tubes (anaerobic culture tubes, 18 × 150 mm, Chemglass Life Science LLC, Vineland, NJ, USA), 1 mL of the fecal slurry was mixed with 14 mL of sterile fermentation medium and no substrate, 100 mg of FOS, or 100 mg of AX was added for control, FOS and AX treatment. Test tubes were tightly sealed with a rubber stopper and autoclave tape and transferred to an incubator at 37 °C. Aliquots from each test tube were taken at the time points 0, 4, 8, 12, and 24 h, mixed with 0.1 mL of stop solution (2 M potassium hydroxide) and stored at −80 °C until analysis.

### 2.5. Determination of Short-Chain Fatty Acids

Short-chain fatty acids in samples at all time points were measured using a modified method of Ahmadi et al. [52]. Samples were thawed and centrifuged at 12,000× *g* for 5 min. The supernatant (300 μL) was mixed with 10 μL of 1 mM of 4-methyl valeric acid as an internal standard. The sample was transferred into a syringe fitted with a 4 mm 45-micron nylon filter and analyzed immediately using HPLC. HPLC was equipped with a Bio-Rad HPLC Organic Acid Analysis Aminex HPX-87H ion exclusion column (300 × 7.8 mm). The column was maintained at 65 °C (0.1 °C by a temperature control unit). The mobile phase consisted of water acidified with sulfuric acid (pH 2.28) with a flow rate of 0.65 mL/min. The solvent delivery system was a Waters 515 HPLC pump equipped with a Waters 717 plus autosampler. A Waters 996 photodiode array detector monitored the eluting compounds at 210 nm. As reference standards, individual and an equimolar mixture of acetic, propionic and butyric acid were used to quantify individual SCFA content.

### 2.6. Analysis of Microbial Composition

Changes in microbial communities during in vitro fermentation with treatments were detected by DNA sequencing of samples at 0 and 24 h time points as previously described [53]. Bacterial DNA in samples at 0 and 24 h were extracted using the OIAamp Fast DNA Stool Mini Kit (Qiagen, Gaithersburg, MD, USA). All DNA samples were diluted to 10 ng/μL after determining the concentration of DNA using NanoDrop^TM^ 1000 Spectrophotometer (Thermo Fisher Scientific, Waltham, WA, USA). The V4 region of 16S rRNA gene in samples was amplified in Eppendorf Mastercycler pro S (Eppendorf, Hamburg, Germany) with AccuPrime Pfx SuperMix (Thermo Fisher Scientific, Waltham, WA, USA) and forward and reverse primers using polymerase chain reaction (PCR). Amplified DNA samples were confirmed by agarose gel electrophoresis and normalized using a SequalPrep Normalization Plate Kit (Thermo Fisher Scientific, Waltham, WA, USA). The pooled DNA library was sequenced with the Illumina MiSeq platform (Illumina, San Diego, CA, USA) based on the 16S-rRNA V4 region [54]. The raw sequencing data from Illumina were processed through Mothur 1.41.1. [55]. For microbial composition analysis, samples from 12 subjects (NW = 6, OO = 6) were used due to a missing sample.

### 2.7. Statistical Analysis

Statistical analyses were performed with SAS 9.4 (SAS Institute Inc., Cary, NC) and R version 3.3.3 (R core Team, Vienna, Austria) for randomized complete block design, where weight classes are confounded with subject blocks. Significant differences among means were determined at a 5% level of significance. The Friedman test was used to compare 3 treatments at each time point in SCFA content and among 2 time points (0 and 24 h) in microbial composition data. To test significant change in the relative abundance of microbiota between 0 and 24 h (time effect), the Wilcoxon signed rank sum test was used. The Mann–Whitney test was used to test significant difference in microbial relative abundance between the NW and OO group (weight effect) at each time point (0 and 24 h).

## 3. Results

### 3.1. Characteristics of Subjects

Subject characteristics including age, BMI, and FBG are shown in Table 1. Six NW (three males, three females) and seven OO (three males, four females), i.e., a total of thirteen, subjects participated in this study. For further information regarding individual subject characteristics in Table 1.

### 3.2. Production of Short-Chain Fatty Acids

**Figure 1:**
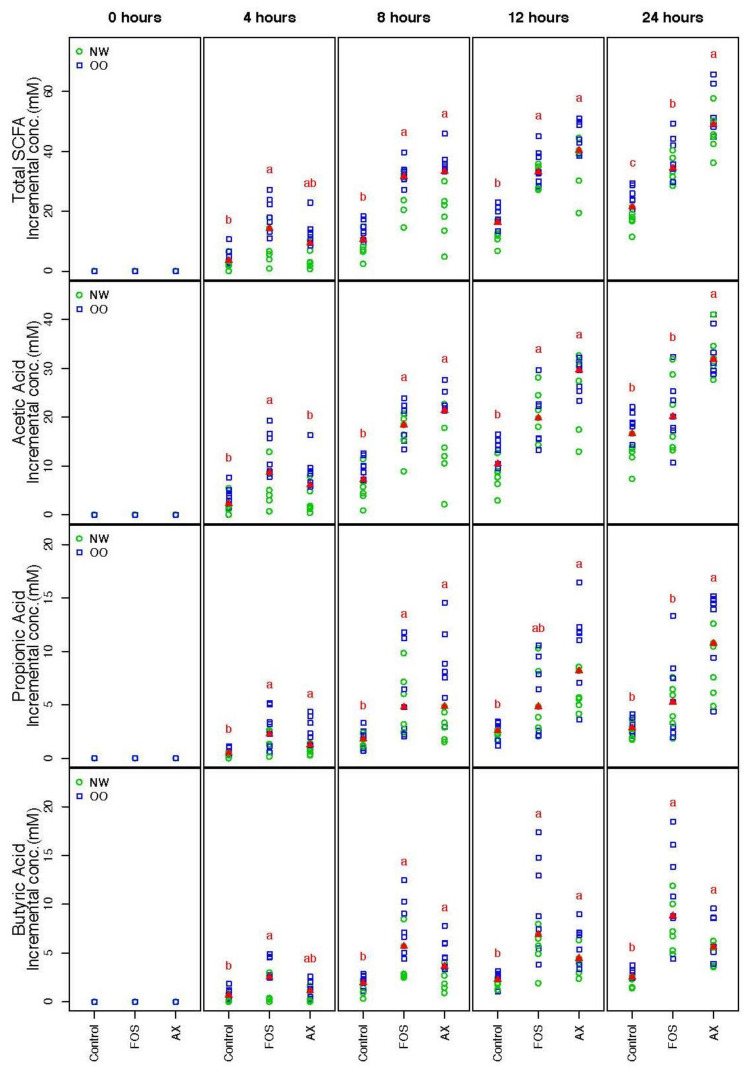
Total and individual short-chain fatty acids (SCFAs) during in vitro fecal fermentation (*n* = 13; NW = 6, OO = 7). Treatments with different letters indicate significant differences at *p* < 0.05 based on the Friedman test and the corresponding multiple testing procedure. The medians are marked with red triangles. The weight classes are labeled by green circles (NW) and blue squares (OO). FOS: fructooligosaccharides, AX: arabinoxylan.

SCFA concentrations from the three different conditions (control, FOS, AX) at time points 0, 4, 8, 12 and 24 h were determined (Figure 1). The trend of the total SCFA production was similar under both FOS and AX treatments; a significant 1.7- to 3.3-fold increase was observed at most time points when compared to the control without fibers. The total SCFA content in the AX treatment was lower at 4 h, but higher after 8 h compared to the FOS treatment.

The individual SCFA production in the AX treatment showed a similar pattern in acetic acid (AA) and propionic acid (PA), where more AA and PA were produced after 8 h when compared to the FOS treatment. However, less butyric acid (BA) was produced in the AX treatment than the FOS treatment at all time points (no significant difference). In general, more AA was produced than PA and BA (AA:PA:BA = 66:17:17).

There were no significant differences in the total and individual SCFA concentrations between males and females. OO class showed a significantly higher value in total SCFA than NW at 0.05 level after adjustment of time and gender effect across all three treatments.

### 3.3. Compositional Changes in Phyla

**Figure 2:**
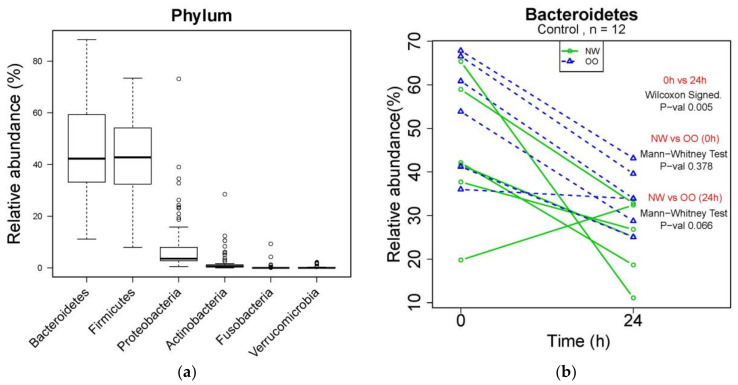
Microbial composition at phylum level during in vitro fecal fermentation. (**a**) The composition of microbiota at phylum level; (**b**) effect of time and weight on composition of Bacteroidetes. The Wilcoxon signed test was used for the effect of time (0 and 24 h), and the Mann–Whitney test was used for the effect of weight (NW and OO) at each time point (0 and 24 h).

Changes in microbial composition during in vitro fecal fermentation by treatments were determined by dual-index high throughput sequencing of the 16 rRNA gene V4 region. At the phylum level, Bacteroidetes, Firmicutes, Proteobacteria, Actinobacteria, Fusobacteria and Verrucomicrobia were identified from bacterial DNA samples (Figure 2a). Bacteroidetes were the most abundant phylum, showing a significant reduction in control at 24 h compared to at 0 h (*p* < 0.05) (Figure 2b). NW showed more drop at 24 h in the relative abundance of Bacteroidetes compared to OO, but with no significant difference. Similar to Figure 2b, the effect of time and weight group in major phyla is shown in Table 2.

After 24 h, the control without fibers significantly decreased Bacteroidetes and increased Proteobacteria, while the FOS treatment significantly increased Bacteroidetes and decreased Firmicutes and Verrucomicrobia (*p* < 0.05) (Table 2). The AX treatment resulted in increased Actinobacteria and decreased Verrucomicrobia after 24 h (*p* < 0.05) (Table 2). There was no significant difference in the relative abundance of any phylum between NW and OO at 24 h. For further information regarding individual changes in Table 2, see Figure S1 and Table S1.

**Figure 3:**
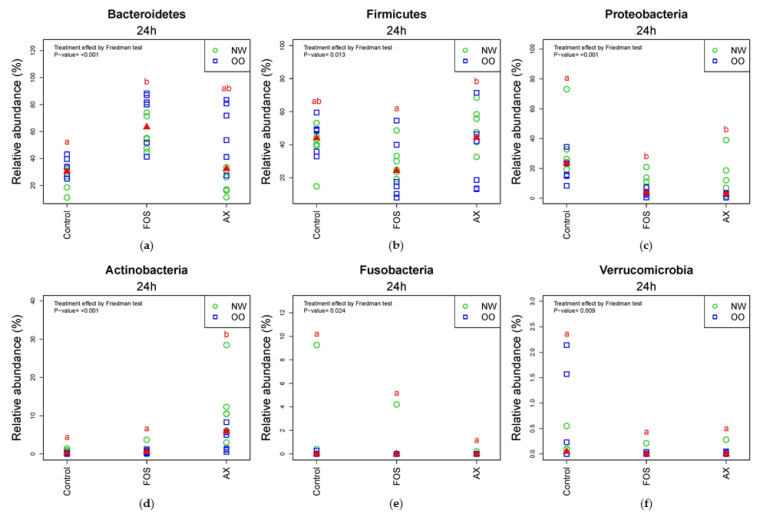
Relative abundance of microbiota at the 24 h time point at phylum level (*n* = 12; NW = 6, OO = 6). (**a**) Bacteroidetes; (**b**) Firmicutes; (**c**) Proteobacteria; (**d**) Actinobacteria; (**e**) Fusobacteria; (**f**) Verrucomicrobia. Treatments with different letters indicate significant differences at *p* < 0.05 based on the Friedman test and the corresponding multiple testing procedure. The medians are marked with red triangles. The weight classes are labeled by green circles (NW) and blue squares (OO). AX: arabinoxylan.

In the comparison of relative abundance of phyla among treatments at 24 h, Bacteroidetes was significantly increased by the FOS treatment compared to the control without fibers (*p* < 0.05) (Figure 3a). OO tended to have more Bacteroidetes than NW in the control and AX treatments at 24 h. Due to a significant drop in the FOS treatment after 24 h, there were less Firmicutes in the FOS treatment than the AX treatment at 24 h (*p* < 0.05) (Figure 3b). Proteobacteria was significantly increased by the control without fibers compared to the FOS and AX treatments (*p* < 0.05) (Figure 3c). NW tended to have more Proteobacteria in all treatments. Actinobacteria showed remarkable increase with the AX treatment compared to other treatments (*p* < 0.05) (Figure 3d). Actinobacteria showed higher relative abundance in NW than in OO in the AX treatment. Fusobacteria (Figure 3e) and Verrucomicrobia (Figure 3f) showed no significant difference among treatments at 24 h.

### 3.4. Compositional Changes in Genera

**Figure 4:**
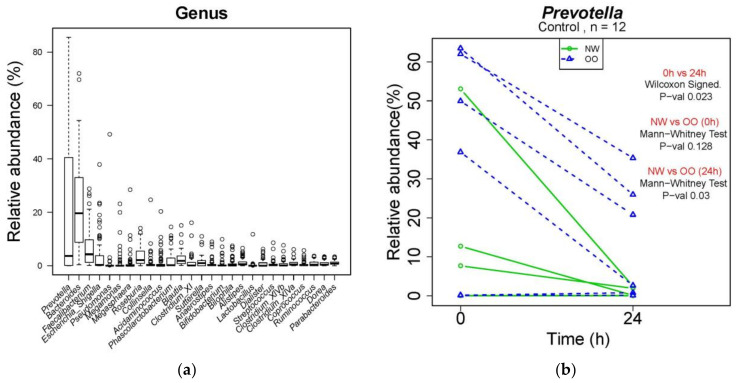
Microbial composition at genus level during in vitro fecal fermentation (*n* = 12; NW = 6, OO = 6). (**a**) The composition of microbiota at genus level; (**b**) effect of time and weight on composition of *Prevotella*. The Wilcoxon signed test was used for the effect of time (0 and 24 h), and the Mann–Whitney test was used for the effect of weight (NW and OO) at each time point (0 and 24 h).

At the genus level, a total of 260 genera were identified from bacterial DNA samples, and the distribution of 27 major genera is shown in Figure 4a. *Prevotella* was the most abundant genus, showing a decrease in the control at 24 h compared to at 0 h (Figure 4b). The relative abundance of *Prevotella* in the control was lower in NW than OO at 24 h, but with no significant difference. Similar to Table 2, the effect of time and weight group at the genus level are shown in Table 3.

In Table 3, all treatments significantly increased *Escherichia/Shigella* and decreased *Faecalibacterium* and *Streptococccus* after 24 h (*p* < 0.05). The FOS treatment significantly reduced *Roseburia*, *Alistipes*, *Megamonas*, *Ruminococcus* and *Lactobacillus*, while the AX treatment resulted in increased *Collinsella* and *Bifidobacterium* and decreased *Sutterella*, *Alistipes*, *Clostridium XIVa* and *b* after 24 h (*p* < 0.05). There was no significant difference in the relative abundance of any genus between NW and OO at 24 h. For further information regarding individual changes in Table 3, see Figure S2 and Table S2.

**Figure 5:**
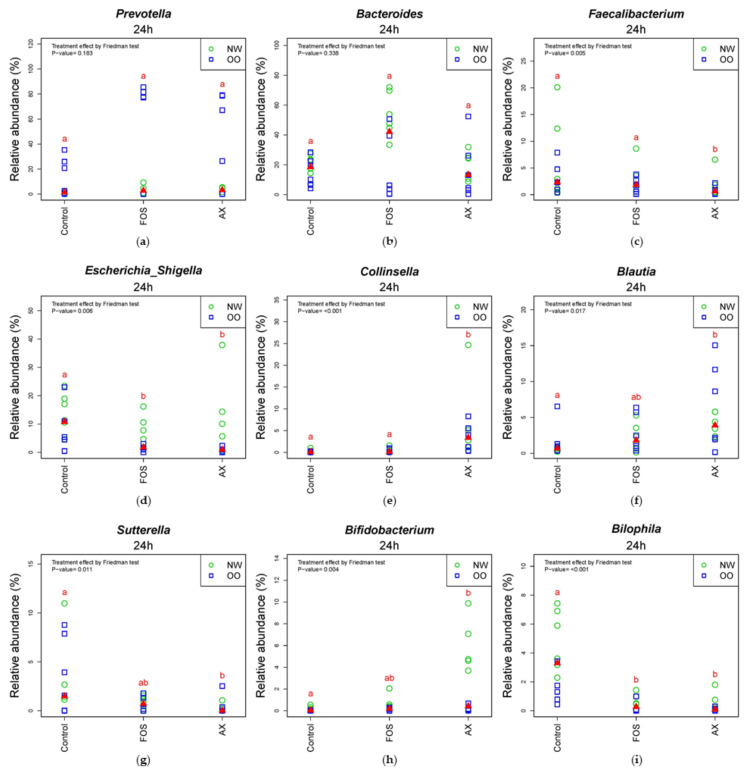

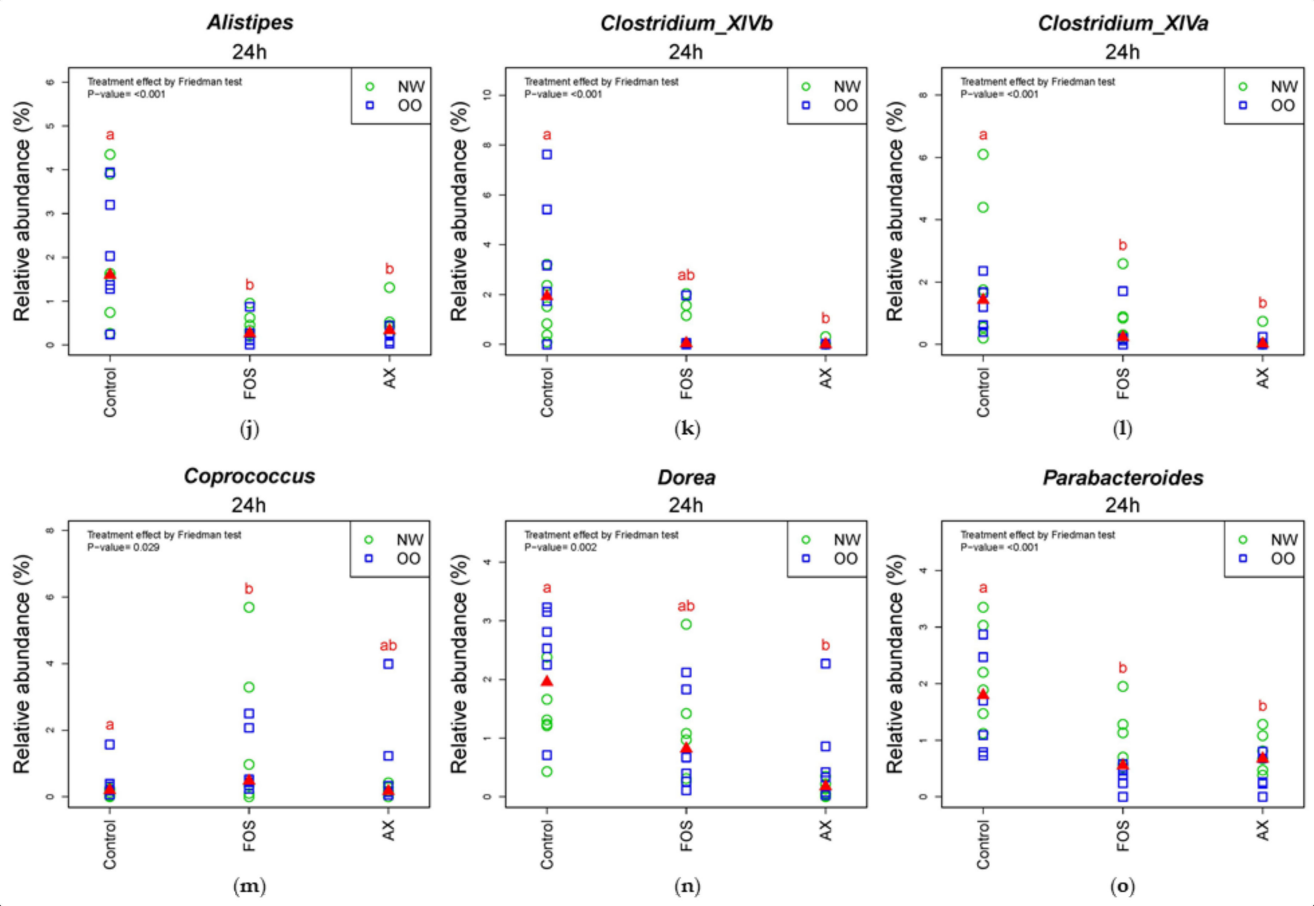
Relative abundance of microbiota at the 24 h time point at genus level (*n* = 12). (**a**) *Prevotella*; (**b**) *Bacteroides*; (**c**) *Faecalibacterium*; (**d**) *Escherichia/Shigella*; (**e**) *Collinsella*; (**f**) *Blautia*; (**g**) *Sutterella*; (**h**) *Bifidobacterium*; (**i**) *Bilophia*; (**j**) *Alistipes*; (**k**) *Clostridium XIVb*; (**l**) *Clostridium XIVa*; (**m**) *Coprococcus*; (**n**) *Dorea*; (**o**) *Parabacteroides*. Treatments with different letters indicate significant differences at *p* < 0.05 based on the Friedman test and the corresponding multiple testing procedure. The medians are marked with red triangles. The weight classes are labeled by green circles (NW) and blue squares (OO). AX: arabinoxylan.

In the comparison of relative abundance of genera among treatments at 24 h (Figure 5), the control without fibers significantly increased *Escherichia/Shigella*, *Parabacteroides*, *Bilophila* and *Dorea* compared to other treatments (*p* < 0.05). The FOS and AX treatments both reduced the abundance of *Alistipes* and *Clostridium XIVa* (*p* < 0.05). The AX treatment significantly increased *Blautia*, *Collinsella* and *Bifidobacterium* and decreased *Faecalibacterium*, *Sutterella* and *Clostridium XIVb*, while *Coprococcus* was increased by the FOS treatment (*p* < 0.05). In the comparison of relative abundance between weight groups at 24 h (Figure 5 and Figure S2), OO tended to have more *Prevotella*, *Dorea* and *Coprococcus* in the control without fibers. NW tended to have more *Bacteroides* and *Parabacteroides* with the FOS treatment, and more *Megamonas* with the AX treatment. With the FOS and AX treatment, the relative abundance of *Escherichia/Shigella* and *Alistipes* was higher in NW than OO. The level of *Bilophila* and *Bifidobacterium* was lower in OO in all treatments. However, these trends between weight groups at 24 h did not show a significant difference (Table 3).

## 4. Discussion

The current study was performed to examine the effect of AX isolated from rice bran on gut microbial composition and SCFA content through in vitro fecal fermentation from NW and OO weight classes. Fructooligosaccharides (FOS) were used as a control with fiber in this study because FOS is a well-established prebiotic [56,57,58].

Acetic acid is the most abundant fermentation product from gut microbes as a net product, and it can be taken up and circulated to peripheral tissues, including muscles, the heart and the brain [31]. It is also used for hepatic cholesterol synthesis and lipogenesis related to body weight and adiposity [59]. In the current study, acetic acid showed the highest concentration among SCFAs and increased time-dependently with the AX treatment. Other studies with the treatment of wheat arabinoxylans [60], feruloylated arabinoxylan oligosaccharides from rice bran (FAXO) [53] and hydrolyzed rice arabinoxylans [61] also showed similar results as the current study by increasing the level of acetic acid the most among SCFAs.

Propionic acid is associated with gluconeogenesis in the liver, cholesterol synthesis inhibition, stimulation of the immune system and pH decrease in the colon [59]. Propionate also increases satiety and reduces appetite, energy uptake, weight gain and other risk factors related to obesity [62]. Propionic acid is produced either via the succinate pathway where succinate is used to make propionic acid or via the acrylate pathway where lactate is used [63,64]. Our results showed that the level of propionic acid in the AX treatment significantly increased at 24 h compared to control and FOS (*p* < 0.05). There were similar findings from the treatment of rice arabinoxylans [61] and FAXO [53], showing a comparable or more significant increase than FOS.

Butyric acid is a main energy source for the colonocytes, and it has a crucial role in improving the immune system, removing oxidative stress in the colon, stimulating the production of mucin and antimicrobial peptides and maintaining epithelial tight-junction integrity, for supporting gut barrier function [65,66]. In addition, it has been implicated for protection from colorectal cancer [65]. Feruloylated arabinoxylan oligosaccharides from rice bran increased butyric acid more than the FOS [53]. However, Rumpagaporn et al. [61] showed that endoxylanase–hydrolysate of rice alkali-extractable arabinoxylan produced less butyric acid than the FOS. Similar to Rumpagaporn et al. [61], we found that the AX treatment increased acetic acid and propionic acid more, but butyric acid less compared to the FOS treatment.

The AX treatment showed a lower fermentation in total and individual SCFA content at 0–4 h compared to the FOS treatment. This can be explained by the structural difference between treatments. In Rosa et al. [67] and Rose et al. [68], rice arabinoxylans had a more branched structure compared to maize arabinoxylans. Since rice arabinoxylans are highly branched, it takes more time for bacterial enzymes to remove arabinose branches and depolymerize xylan for subsequent fermentation. Kaur et al. [69] compared SCFA production of different dietary fibers using in vitro upper gastrointestinal digestion and human fecal fermentation for 48 h. They found that FOS showed the highest production in total SCFA at 4 h, with rapid fermentation at an early period. Maize bran AX showed the lowest concentration of SCFA at 4 h, with a slow fermenting rate at the early period, but their fermentation rate increased at 8 h, with rapid fermentation at a later period. Their results are consistent with our results for rice bran AX.

Between weight classes, OO had a higher total SCFA level than NW (*p* < 0.05) after adjusting the effects of time and gender in all treatments. Our results are in line with previous studies [70]. Kim et al. [70] combined the studies with SCFA levels of obese and nonobese individuals using a meta-analysis, and the results showed that obese individuals had significantly higher acetic acid, propionic acid and butyric acid concentration in fecal samples than nonobese individuals.

The major phyla after in vitro fecal fermentation with treatments were Bacteroidetes, Firmicutes, Proteobacteria, Actinobacteria, Fusobacteria and Verrucomicrobia. Bacteroidetes and Firmicutes are the most abundant phyla in the gut microbiota, accounting for more than 90% [71]. There have been many studies which have found that the Bacteroidetes/Firmicutes ratio is associated with obesity [51,72,73]. Obese individuals tend to have decreased Bacteroidetes and increased Firmicutes compared to normal-weight groups [74]. However, the association between the ratio and obesity has been controversial. Some studies found no significant differences in the Bacteroidetes/Firmicutes ratio between lean and obese people, and others showed opposite trends [23,75,76]. Duncan et al. [25] found that the Bacteroidetes/Firmicutes ratio does not determine human obesity. The Bacteroidetes/Firmicutes ratio can be very different among subjects due to many factors, such as diet, lifestyle and energy expenditure activity, so it is hard to use the ratio as an indicator of obesity [77]. In this study, there was no significant difference in Bacteroidetes and Firmicutes level between NW and OO. In addition, OO showed a relatively higher Bacteroidetes level than NW in AX at 24 h.

Proteobacteria and Actinobacteria are the major subdominant phyla in the gut microbiota, followed by the dominant phyla, Bacteroidetes and Firmicutes [78]. Chakraborti [78] reported that obese individuals showed a higher abundance of Actinobacteria. However, a recent review reported that Actinobacteria have an important role in developing and maintaining gut homeostasis, especially with an Actinobacteria genus, Bifidobacterium, a well-known probiotic [79]. The current study showed different results than Chakraborti [78]. In the current study, there was no significant difference in the abundance of Actinobacteria between NW and OO. In addition, AX significantly increased the abundance of Actinobacteria compared to FOS at 24 h.

*Blautia* is a genus that has shown a significant negative correlation with many diseases related to inflammation and obesity [80]. *Blautia* had a lower abundance in diabetic patients [81]. In another study, *Blautia* was more abundant in the gut microbiota of women from the effective weight loss group compared to the ineffective weight loss group [82]. *Sutterella* is a genus in Proteobacteria, and it was significantly elevated in obese individuals in Finegold [83] and Lv et al. [84]. *Sutterella* is also linked to human diseases, including inflammatory bowel disease, autism and Down syndrome [85]. In our study, AX significantly increased *Blautia* and decreased *Sutterella*, suggesting a possible beneficial impact on host health. However, there are still controversial results associated with many diseases, and the causal relationship with diseases is not extensively studied, so further studies are needed to confirm their impact on host health [70,80].

Traditionally, *Bifidobacterium* has been considered a beneficial bacterium [33]. *Bifidobacterium* has health-beneficial effects via many different pathways [86,87]. It reduces lipopolysaccharide levels, improves the tight-junction integrity of epithelial cells and prevents inflammatory diseases [88]. AX in the current study significantly increased *Bifidobacterium* level compared to control (*p* < 0.05), while NW showed relatively higher *Bifidobacterium* than the OO group. AX has been reported to be a preferential substrate for *Bifidobacterium* by several in vitro and in vivo studies [40,42,89,90,91,92], and our results support this model.

## 5. Conclusions

In conclusion, the present study showed that the in vitro fecal fermentation of rice bran AX increased the total SCFA content and modified the microbiota in fecal samples from both weight classes, NW and OO. There was a significant difference in total SCFA concentration between NW and OO, but both weight classes showed similar trends in fermentation patterns with AX treatment. This suggests that rice bran arabinoxylan may beneficially impact microbial gut health in obesity through prebiotic activities. However, further studies are needed to investigate the interaction with the host health due to the limitations of the current study.

## Figures and Tables

**Table 1:** Subject characteristics.

	All (*n* = 13)	Normal Weight		Overweight/Obese	
		Male (*n* = 3)	Female (*n* = 3)	Male (*n* = 3)	Female (*n* = 4)
Age (years)	27.5 ± 7.1	29.0 ± 8.0	24.3 ± 3.1	24.7 ± 2.3	31.0 ± 10.7
BMI (kg/m^2^)	28.3 ± 6.3	23.1 ± 0.2	21.8 ± 0.8	32.3 ± 3.5	33.9 ± 5.0
FBG (mg/dL)	93.1 ± 5.3	91.5 ± 5.6	89.7 ± 4.0	94.7 ± 8.3	95.6 ± 3.4

Data are expressed as mean ± standard deviation (SD). BMI: body mass index, FBG: fasting blood glucose.

**Table 2:** The effect of time and weight group in the major phyla.

Microbiota	Treatment	Change in 24 h Relative Abundance (%)	Wilcoxon Signed Test (0 and 24 h) adj. *p*-Value ^1^	Mann–Whitney Test (NW and OO) at 24 h adj. *p*-Value ^1^
	Control	−20	**0.016**	0.236
Bacteroidetes	FOS	20.5	**0.015**	0.414
	AX	−6.6	0.420	0.235
	Control	−3.3	0.606	0.606
Firmicutes	FOS	−23.2	**0.015**	0.447
	AX	−3.9	0.293	0.414
	Control	22.7	**0.015**	0.278
Proteobacteria	FOS	3.3	0.227	0.236
	AX	3.7	0.950	0.236
	Control	−0.2	0.278	0.395
Actinobacteria	FOS	0.1	1.000	0.684
	AX	7.1	**0.015**	0.236
	Control	0.7	0.211	0.414
Fusobacteria	FOS	0.3	1.000	0.395
	AX	−0.1	0.130	0.560
	Control	0.2	0.324	0.684
Verrucomicrobia	FOS	−0.3	**0.015**	1.000
	AX	−0.2	**0.024**	1.000

^1^ adjusted *p*-value by Holm’s multiple testing procedure. The significant effect is denoted by bold in *p*-values at 0.05. FOS: fructooligosaccharides, AX: arabinoxylan.

**Table 3:** The effect of time and weight group at the genus level.

Taxon (Phylum)	Treatment	Change in 24 h Relative Abundance (%)	Wilcoxon Signed Test (0 and 24 h) adj. *p*-Value ^1^	Mann–Whitney Test (NW and OO) at 24 h adj. *p*-Value ^1^
*Prevotella* (Bacteroidetes)	Control	−16.3	0.062	0.310
	FOS	8	0.945	0.476
	AX	−0.2	0.660	0.476
*Bacteroides* (Bacteroidetes)	Control	−4.6	0.606	0.734
	FOS	15.5	0.066	0.310
	AX	−3.4	0.342	0.734
*Faecalibacterium* (Firmicutes)	Control	−6.9	**0.019**	0.948
	FOS	−7.9	**0.019**	0.681
	AX	−10.5	**0.019**	0.589
*Roseburia* (Firmicutes)	Control	−3.5	**0.024**	0.505
	FOS	−3.4	**0.042**	0.734
	AX	−1.9	0.227	0.505
*Escherichia/Shigella* (Proteobacteria)	Control	10.8	**0.019**	0.455
	FOS	4	**0.019**	0.310
	AX	6	**0.020**	0.310
*Blautia* (Firmicutes)	Control	−0.9	0.060	0.476
	FOS	0.2	0.993	0.872
	AX	3	0.102	0.911
*Phascolarctobacterium* (Firmicutes)	Control	1.2	0.060	0.911
	FOS	−0.4	0.863	0.911
	AX	−1	0.879	0.911
*Sutterella* (Proteobacteria)	Control	1.9	0.192	0.832
	FOS	−0.3	0.712	0.872
	AX	−1	**0.042**	0.734
*Alistipes* (Bacteroidetes)	Control	0.8	0.186	0.948
	FOS	−1.2	**0.019**	0.439
	AX	−1.2	**0.020**	0.310
*Megamonas* (Firmicutes)	Control	−0.7	**0.030**	0.476
	FOS	−0.3	**0.019**	0.455
	AX	2.8	0.965	0.439
*Collinsella* (Actinobacteria)	Control	0	0.392	0.728
	FOS	0	0.863	0.942
	AX	4.7	**0.019**	0.872
*Parabacteroides* (Bacteroidetes)	Control	0.9	**0.030**	0.505
	FOS	−0.3	0.203	0.310
	AX	−0.4	0.078	0.476
*Clostridium_XlVa* (Firmicutes)	Control	1	0.165	0.832
	FOS	−0.2	0.203	0.455
	AX	−0.8	**0.021**	0.728
*Bilophila* (Proteobacteria)	Control	3.2	**0.019**	0.310
	FOS	0.3	0.079	0.408
	AX	0.1	0.966	0.439
*Dorea* (Firmicutes)	Control	1.5	**0.019**	0.439
	FOS	0.4	0.345	0.851
	AX	−0.3	0.481	0.455
*Ruminococcus* (Firmicutes)	Control	−0.5	0.294	0.851
	FOS	−0.7	**0.030**	0.485
	AX	−0.3	0.446	0.505
*Clostridium_XlVb* (Firmicutes)	Control	2.1	**0.021**	0.589
	FOS	0.3	0.818	0.681
	AX	−0.3	**0.021**	0.753
*Streptococcus* (Firmicutes)	Control	−1.5	**0.024**	0.851
	FOS	−0.7	**0.019**	0.948
	AX	−0.5	**0.036**	0.911
*Bifidobacterium* (Actinobacteria)	Control	−0.1	0.894	0.310
	FOS	0.2	0.481	0.439
	AX	2.4	**0.027**	0.439
*Coprococcus* (Firmicutes)	Control	−0.1	0.927	0.439
	FOS	1	0.085	0.948
	AX	0.2	0.930	0.911
*Lactobacillus* (Firmicutes)	Control	0	0.481	0.942
	FOS	−0.1	**0.024**	1.000
	AX	0.9	0.993	0.525

^1^ adjusted *p*-value by Holm’s multiple testing procedure. The significant effect is denoted by bold in *p*-values at 0.05. FOS: fructooligosaccharides, AX: arabinoxylan.

## Data Availability

The data presented in this study are available on request from the corresponding author.

## Supplementary Materials

The following are available online at https://www.mdpi.com/article/10.3390/nu13062052/s1, Figure S1: Effect of time and weight on composition of major phyla, Figure S2: Effect of time and weight on composition of major genera, Table S1: The effect of time in each weight group at the phylum level, Table S2: The effect of time in each weight group at the genus level.
