# Supplementary material for: In Vitro Fecal Fermentation Patterns of Arabinoxylan from Rice Bran on Fecal Microbiota from Normal-Weight and Overweight/Obese Subjects
Source: Nutrients. 2021 Jun 15;13(6):2052. doi: 10.3390/nu13062052 (PMC8232586; doi:10.3390/nu13062052)
Supplement: Supplementary file 1 [file nutrients-13-02052-s001.zip › nutrients-1213360-supplementary.pdf]

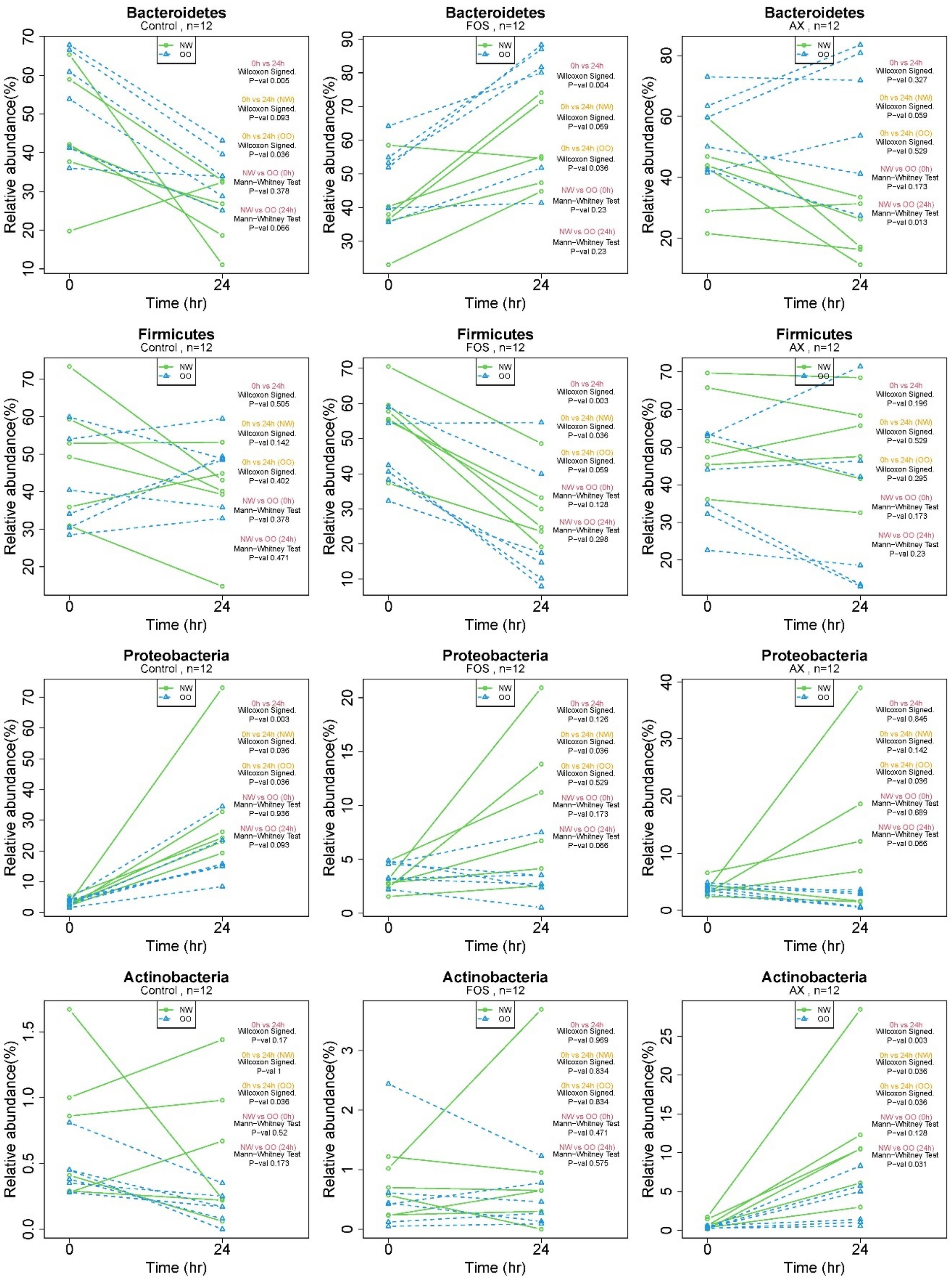

Supplementary Figure S1. Effect of time and weight on composition of major phyla. Wilcoxon signed test was used for the effect of time (0 and 24 h) and Mann-Whitney test was used for the effect of weight (NW and OO) at each time point (0 and 24 h).

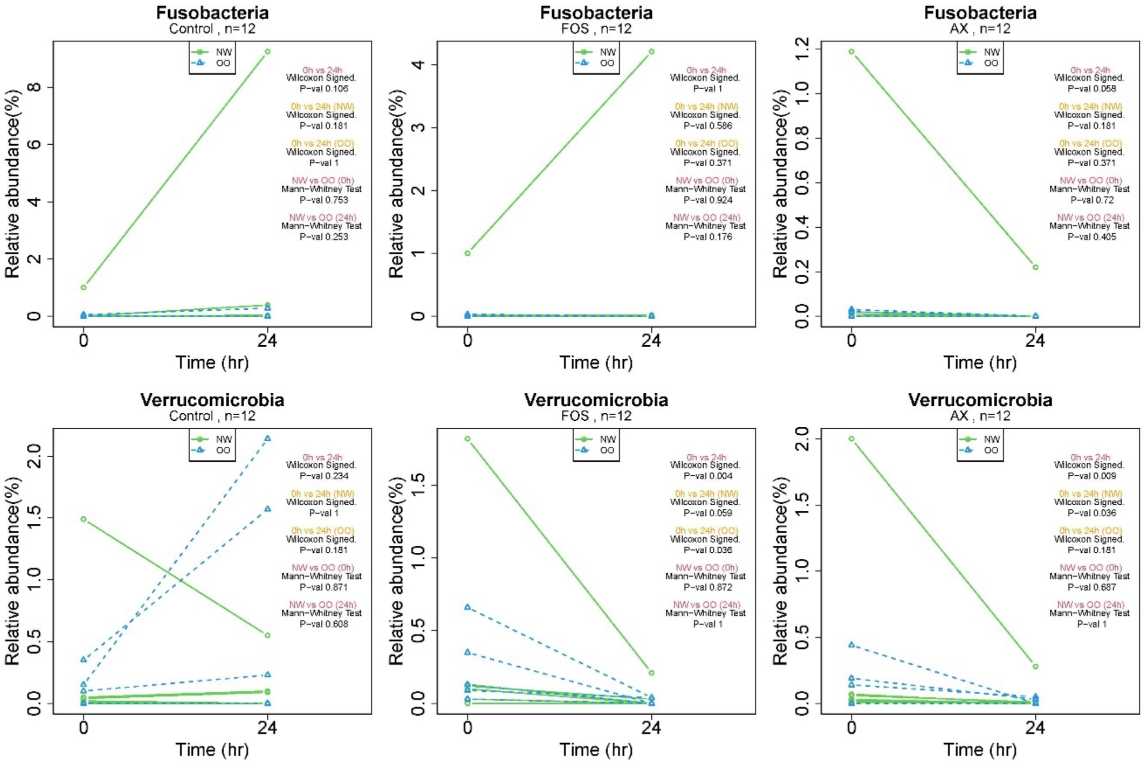

Supplementary Figure S1. *Cont.*

Supplementary Table S1. The effect of time in each weight group at the phylum level.

Microbiota	Treatment	Change in 24 h Relative Abundance (%)	0 and 24 h adj.P-Value[1]	0 and 24 h (NW) adj.P-Value[1]	0 and 24 h (OO) adj.P-Value[1]
Bacteroidetes	Control	-20 (NW:-19.8, OO:-20.3)	**0.016**	0.186	0.093
	FOS	20.5 (NW:19.2, OO:21.7)	**0.015**	0.133	0.093
	AX	-6.6 (NW:-18, OO:4.7)	0.420	0.133	0.595
Firmicutes	Control	-3.3 (NW:-11.1, OO:4.6)	0.606	0.232	0.517
	FOS	-23.2 (NW:-26.1, OO:-20.3)	**0.015**	0.130	0.133
	AX	-3.9 (NW:-1.9, OO:-5.9)	0.293	0.680	0.483
Proteobacteria	Control	22.7 (NW:30, OO:15.5)	**0.015**	0.130	0.093
	FOS	3.3 (NW:7, OO:-0.4)	0.227	0.130	0.595
	AX	3.7 (NW:9.4, OO:-2)	0.950	0.232	0.093
Actinobacteria	Control	-0.2 (NW:-0.2, OO:-0.3)	0.278	1.000	0.093
	FOS	0.1 (NW:0.4, OO:-0.2)	1.000	0.938	0.883
	AX	7.1 (NW:11, OO:3.3)	**0.015**	0.130	0.093
Fusobacteria	Control	0.7 (NW:1.4, OO:0)	0.211	0.251	1.000
	FOS	0.3 (NW:0.5, OO:0)	1.000	0.703	0.514
	AX	-0.1 (NW:-0.2, OO:0)	0.130	0.251	0.514
Verrucomicrobia	Control	0.2 (NW:-0.1, OO:0.6)	0.324	1.000	0.326
	FOS	-0.3 (NW:-0.3, OO:-0.2)	**0.015**	0.133	0.093
	AX	-0.2 (NW:-0.3, OO:-0.1)	**0.024**	0.130	0.326

[1] **Wilcoxon signed test adjusted p-value by Holm's multiple testing procedure. The significant effect is denoted by bold in p-values at 0.05.** FOS: fructooligosaccharides, AX: arabinoxylan.

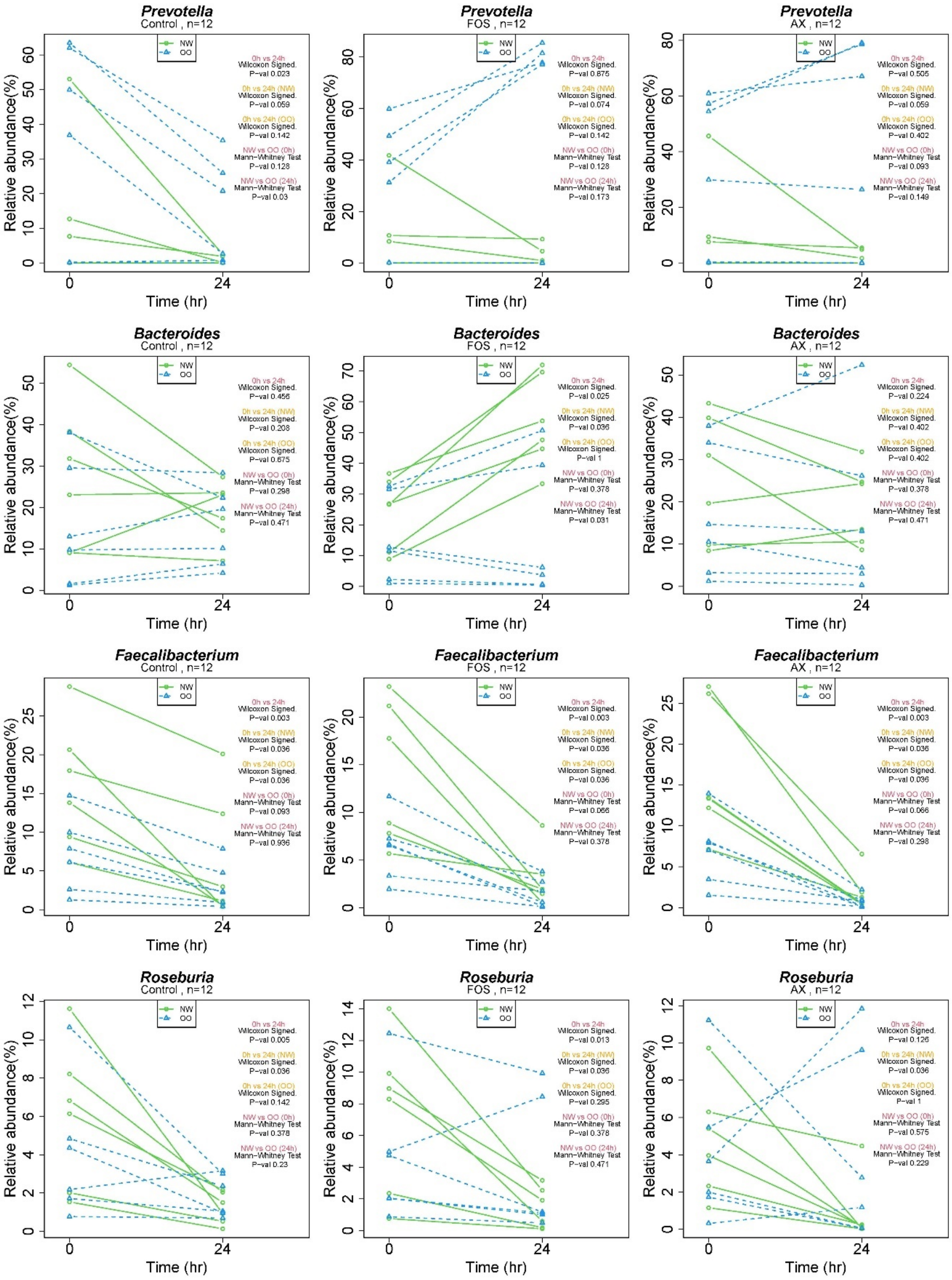

Supplementary Figure S2. Effect of time and weight on composition of major genera. Wilcoxon signed test for the effect of time and Mann-Whitney test for the effect of weight at each time point were used.

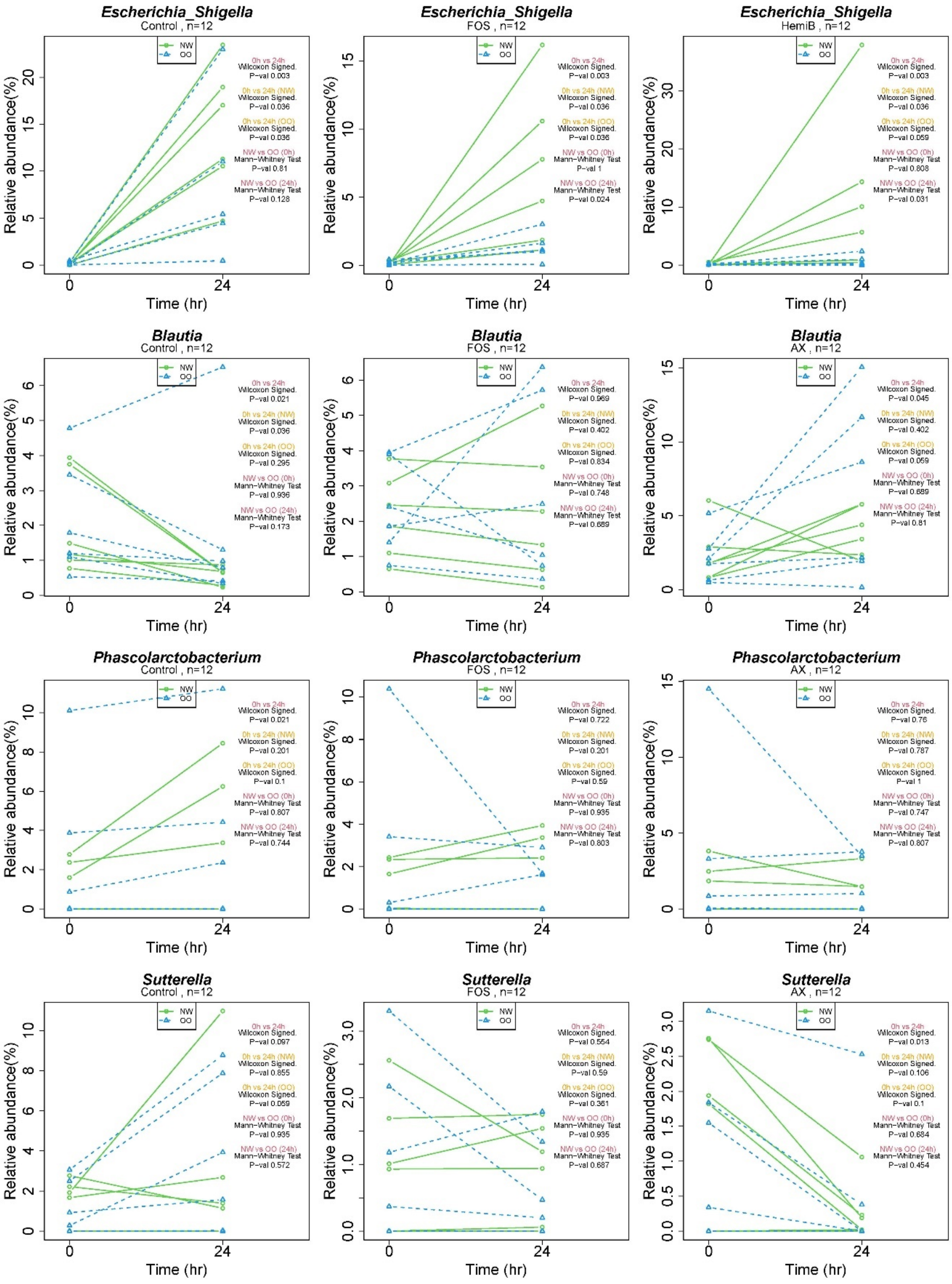

Supplementary Figure S2. *Cont.*

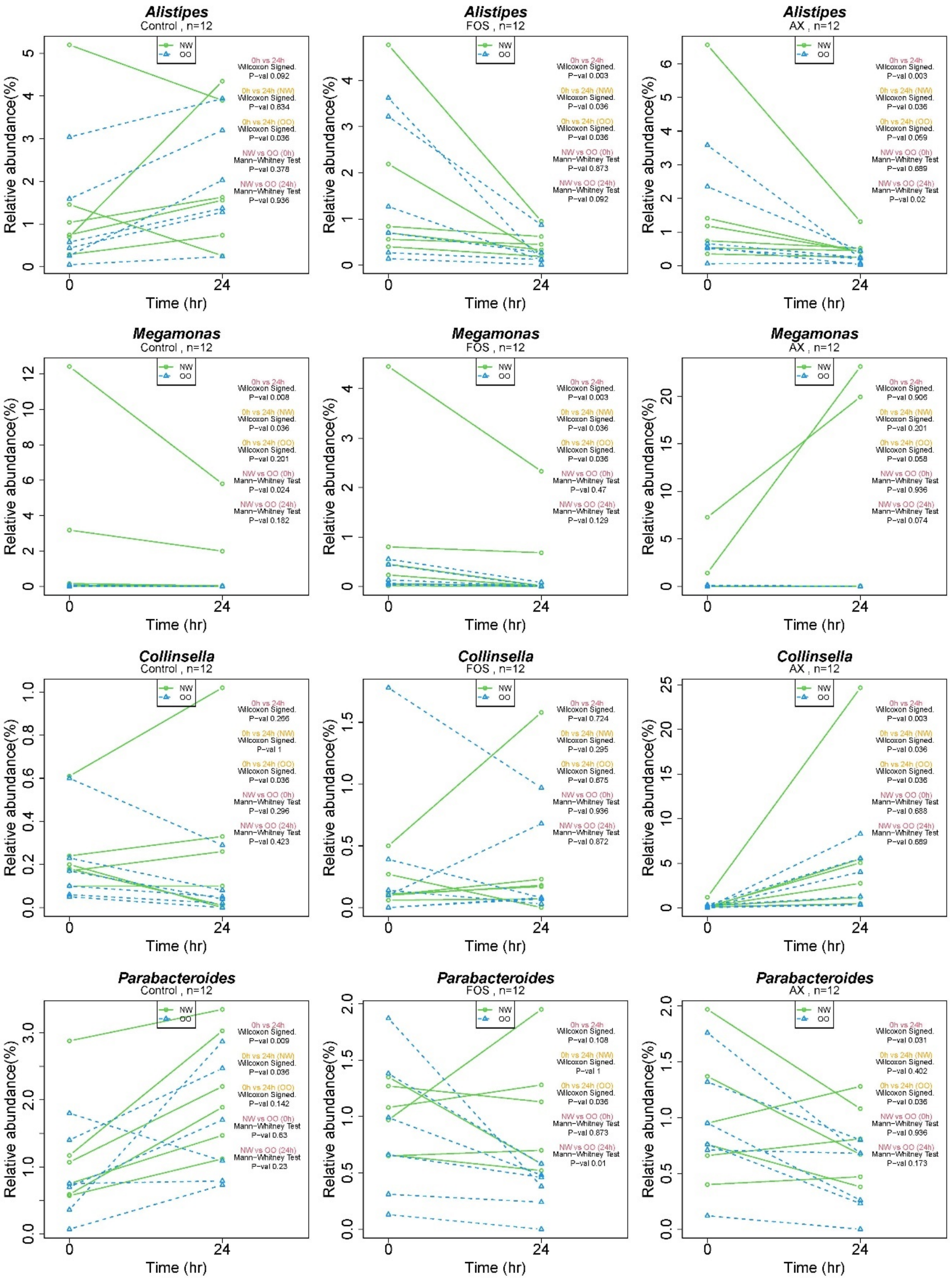

Supplementary Figure S2. *Cont.*

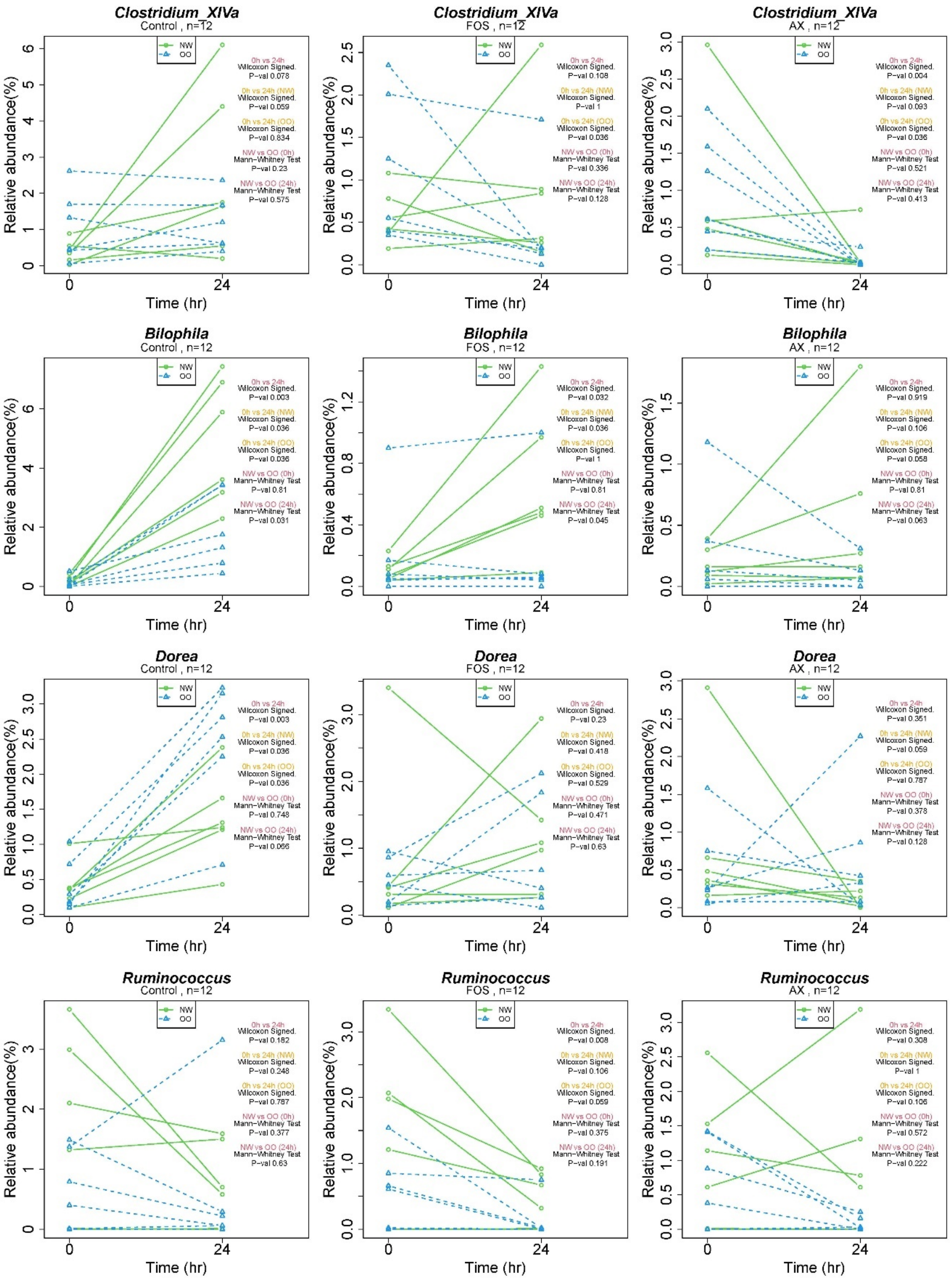

Supplementary Figure S2. *Cont.*

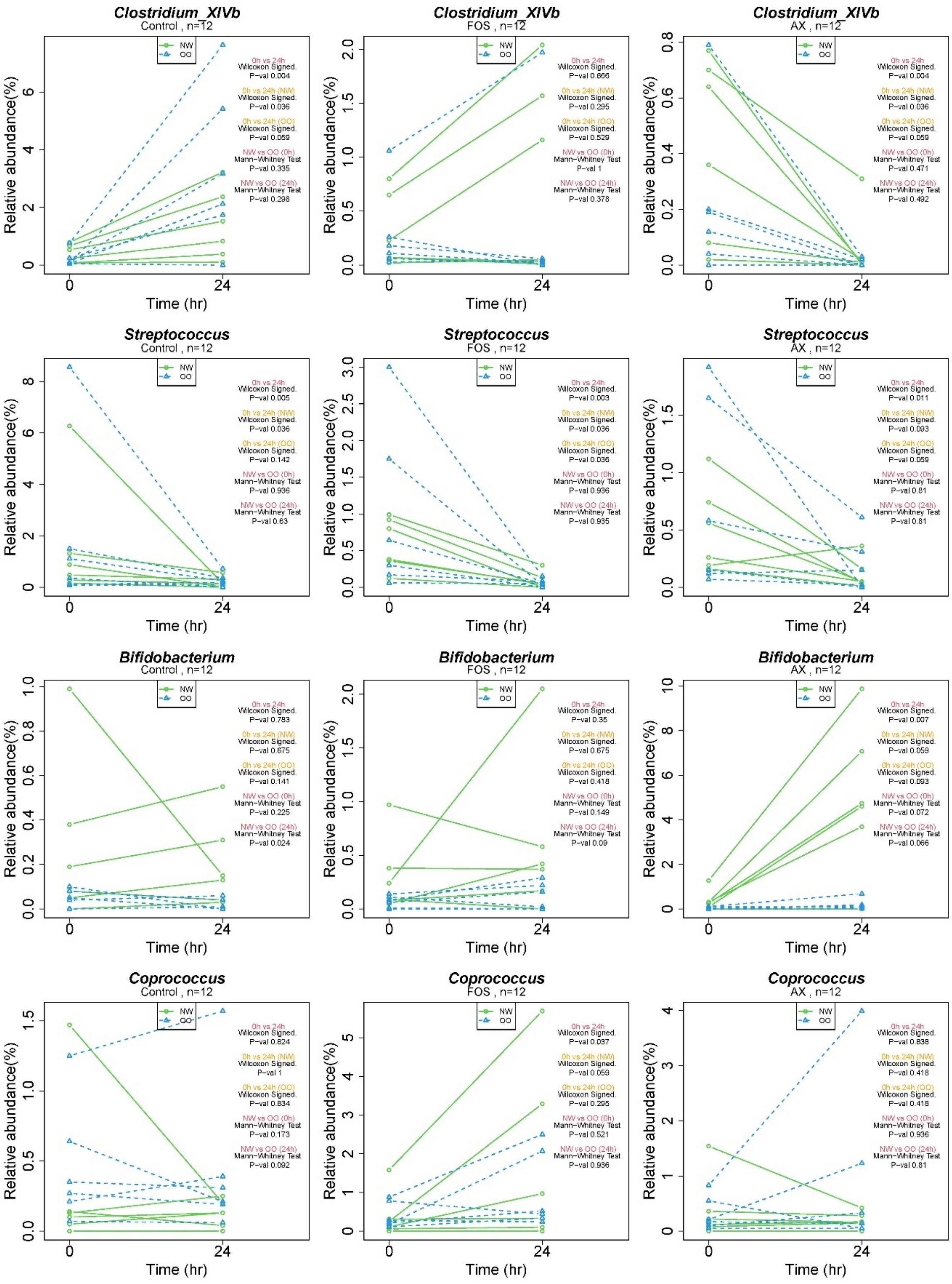

Supplementary Figure S2. *Cont.*

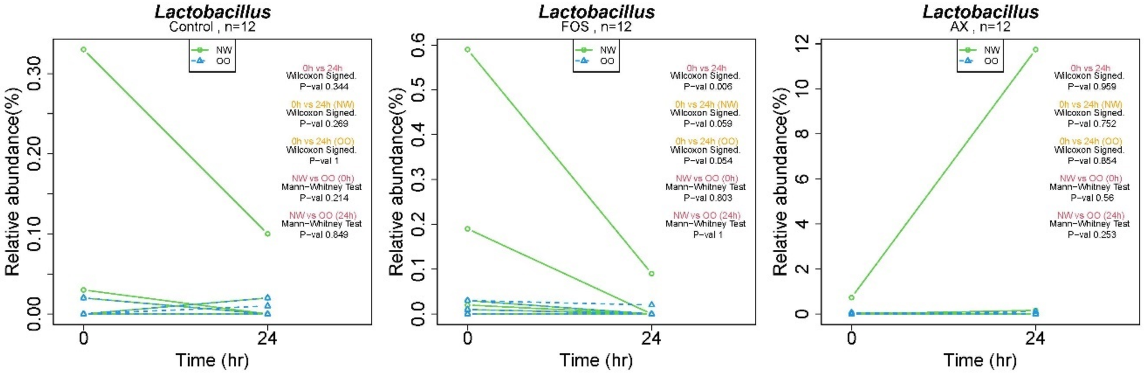

Supplementary Figure S2. *Cont.*

Supplementary Table S2. The effect of time in each weight group at the genus level.

Taxon (Phylum)	Treatment	Change in 24 h Relative Abundance (%)	0 and 24 h adj.P-Value[1]	0 and 24 h (NW) adj.P-Value[1]	0 and 24 h (OO) adj.P-Value[1]
Prevotella (Bacteroidetes)	Control	-16.3 (NW:-11.5, OO:-21.1)	0.062	0.154	0.267
	FOS	8 (NW:-7.7, OO:23.7)	0.945	0.187	0.267
	AX	-0.2 (NW:-8.5, OO:8)	0.660	0.154	0.603
Bacteroides (Bacteroidetes)	Control	-4.6 (NW:-8.8, OO:-0.4)	0.606	0.366	0.868
	FOS	15.5 (NW:29.5, OO:1.6)	0.066	0.122	1.000
	AX	-3.4 (NW:-6.4, OO:-0.4)	0.342	0.552	0.603
Faecalibacterium (Firmicutes)	Control	-6.9 (NW:-9.9, OO:-4)	**0.019**	0.122	0.153
	FOS	-7.9 (NW:-11.1, OO:-4.7)	**0.019**	0.122	0.153
	AX	-10.5 (NW:-14.7, OO:-6.3)	**0.019**	0.122	0.153
Roseburia (Firmicutes)	Control	-3.5 (NW:-4.8, OO:-2.2)	**0.024**	0.122	0.267
	FOS	-3.4 (NW:-6, OO:-0.8)	**0.042**	0.122	0.478
	AX	-1.9 (NW:-4, OO:0.2)	0.227	0.122	1.000
Escherichia/Shigella (Proteobacteria)	Control	10.8 (NW:14.2, OO:7.3)	**0.019**	0.122	0.153
	FOS	4 (NW:6.9, OO:1)	**0.019**	0.122	0.153
	AX	6 (NW:11.4, OO:0.6)	**0.020**	0.122	0.159
Blautia (Firmicutes)	Control	-0.9 (NW:-1.5, OO:-0.4)	0.060	0.122	0.478
	FOS	0.2 (NW:0, OO:0.4)	0.993	0.552	0.965
	AX	3 (NW:1.6, OO:4.5)	0.102	0.552	0.159
Phascolarctobacterium (Firmicutes)	Control	1.2 (NW:1.9, OO:0.5)	0.060	0.362	0.231
	FOS	-0.4 (NW:0.5, OO:-1.3)	0.863	0.362	0.783
	AX	-1 (NW:-0.3, OO:-1.7)	0.879	0.924	1.000
Sutterella (Proteobacteria)	Control	1.9 (NW:1.3, OO:2.6)	0.192	0.949	0.159
	FOS	-0.3 (NW:-0.1, OO:-0.5)	0.712	0.759	0.573
	AX	-1 (NW:-1.3, OO:-0.7)	0.042	0.220	0.231
Alistipes (Bacteroidetes)	Control	0.8 (NW:0.5, OO:1)	0.186	0.938	0.153
	FOS	-1.2 (NW:-1.1, OO:-1.3)	**0.019**	0.122	0.153
	AX	-1.2 (NW:-1.2, OO:-1.1)	**0.020**	0.122	0.159
Megamonas (Firmicutes)	Control	-0.7 (NW:-1.4, OO:0)	**0.030**	0.122	0.354
	FOS	-0.3 (NW:-0.5, OO:-0.2)	**0.019**	0.122	0.153
	AX	2.8 (NW:5.7, OO:0)	0.965	0.362	0.159

Collinsella (Actinobacteria)	Control	0 (NW:0, OO:-0.1)	0.392	1.000	0.153
	FOS	0 (NW:0.2, OO:-0.1)	0.863	0.460	0.868
	AX	4.7 (NW:6.3, OO:3.2)	**0.019**	0.122	0.153
Parabacteroides (Bacteroidetes)	Control	0.9 (NW:1, OO:0.8)	**0.030**	0.122	0.267
	FOS	-0.3 (NW:0, OO:-0.5)	0.203	1.000	0.153
	AX	-0.4 (NW:-0.2, OO:-0.5)	0.078	0.552	0.153
Clostridium_XlVa (Firmicutes)	Control	1 (NW:2, OO:0)	0.165	0.154	0.965
	FOS	-0.2 (NW:0.3, OO:-0.7)	0.203	1.000	0.153
	AX	-0.8 (NW:-0.7, OO:-1)	**0.021**	0.220	0.153
Bilophila (Proteobacteria)	Control	3.2 (NW:4.7, OO:1.7)	**0.019**	0.122	0.153
	FOS	0.3 (NW:0.6, OO:0)	0.079	0.122	1.000
	AX	0.1 (NW:0.3, OO:-0.2)	0.966	0.220	0.159
Dorea (Firmicutes)	Control	1.5 (NW:1, OO:2)	**0.019**	0.122	0.153
	FOS	0.4 (NW:0.4, OO:0.4)	0.345	0.555	0.739
	AX	-0.3 (NW:-0.7, OO:0.2)	0.481	0.154	0.965
Ruminococcus (Firmicutes)	Control	-0.5 (NW:-1, OO:0)	0.294	0.427	0.965
	FOS	-0.7 (NW:-1, OO:-0.5)	**0.030**	0.220	0.159
	AX	-0.3 (NW:0, OO:-0.6)	0.446	1.000	0.232
Clostridium_XlVb (Firmicutes)	Control	2.1 (NW:1, OO:3.1)	**0.021**	0.122	0.159
	FOS	0.3 (NW:0.5, OO:0.1)	0.818	0.460	0.739
	AX	-0.3 (NW:-0.4, OO:-0.2)	**0.021**	0.122	0.159
Streptococcus (Firmicutes)	Control	-1.5 (NW:-1.4, OO:-1.7)	**0.024**	0.122	0.267
	FOS	-0.7 (NW:-0.5, OO:-0.9)	**0.019**	0.122	0.153
	AX	-0.5 (NW:-0.4, OO:-0.6)	**0.036**	0.220	0.159
Bifidobacterium (Actinobacteria)	Control	-0.1 (NW:-0.1, OO:0)	0.894	0.841	0.267
	FOS	0.2 (NW:0.3, OO:0)	0.481	0.841	0.605
	AX	2.4 (NW:4.6, OO:0.2)	**0.027**	0.154	0.231
Coprococcus (Firmicutes)	Control	-0.1 (NW:-0.2, OO:0)	0.927	1.000	0.965
	FOS	1 (NW:1.3, OO:0.6)	0.085	0.154	0.478
	AX	0.2 (NW:-0.2, OO:0.6)	0.930	0.555	0.605
Lactobacillus (Firmicutes)	Control	0 (NW:0, OO:0)	0.481	0.454	1.000
	FOS	-0.1 (NW:-0.1, OO:0)	**0.024**	0.154	0.159
	AX	0.9 (NW:1.8, OO:0)	0.993	0.923	0.974

[1] **Wilcoxon signed test adjusted p-value by Holm's multiple testing procedure. The significant effect is denoted by bold in p-values at 0.05.** FOS: fructooligosaccharides, AX: arabinoxylan.
